# Transcriptomic analysis reveals the roles of gibberellin-regulated genes and transcription factors in regulating bolting in lettuce (*Lactuca sativa* L.)

**DOI:** 10.1371/journal.pone.0191518

**Published:** 2018-02-07

**Authors:** Xueying Liu, Shanshan Lv, Ran Liu, Shuangxi Fan, Chaojie Liu, Renyi Liu, Yingyan Han

**Affiliations:** 1 Plant Science and Technology College, Beijing University of Agriculture/New Technological Laboratory in Agriculture Application in Beijing/ Key Laboratory of Urban Agriculture (North) of Ministry of Agriculture P. R. China, Beijing, China; 2 Shanghai Center for Plant Stress Biology, Shanghai Institutes for Biological Sciences, Chinese Academy of Sciences, Shanghai, China; Universidade de Lisboa Instituto Superior de Agronomia, PORTUGAL

## Abstract

A cool temperature is preferred for lettuce cultivation, as high temperatures cause premature bolting. Accordingly, exploring the mechanism of bolting and preventing premature bolting is important for agriculture. To explore this relationship in depth, morphological, physiological, and transcriptomic analyses of the bolting-sensitive line S39 at the five-leaf stage grown at 37°C were performed in the present study. Based on paraffin section results, we observed that S39 began bolting on the seventh day at 37°C. During bolting in the heat-treated plants, GA3 and GA4 levels in leaves and the indoleacetic acid (IAA) level in the stem reached a maximum on the sixth day, and these high contents were maintained. Additionally, bolting begins in the fifth day after GA3 treatment in S39 plants, GA3 and GA4 increased and then decreased, reaching a maximum on the fourth day in leaves. Similarly, IAA contents reached a maximum in the stem on the fifth day. No bolting was observed in the control group grown at 25°C, and significant changes were not observed in GA3 and GA4 levels in the controls during the observation period. RNA-sequencing data implicated transcription factors (TFs) in regulating bolting in lettuce, suggesting that the high GA contents in the leaves and IAA in the stem promote bolting. TFs possibly modulate the expression of related genes, such as those encoding hormones, potentially regulating bolting in lettuce. Compared to the control group, 258 TFs were identified in the stem of the treatment group, among which 98 and 156 were differentially up- and down-regulated, respectively; in leaves, 202 and 115 TFs were differentially up- and down-regulated, respectively. Significant changes in the treated group were observed for C2H2 zinc finger, AP2-EREBP, and WRKY families, indicating that these TFs may play important roles in regulating bolting.

## Introduction

Lettuce (*Lactuca sativa* L.), which originated on the Mediterranean coast, is widely grown in China, and cool temperatures are preferred for its cultivation. High temperatures induce a transition from vegetative to reproductive growth, and an extended period at high temperatures causes premature bolting, which seriously affects the quality and economic value of the product.

Bolting is a complex process that is subject to auto-feedback and is affected by several factors, like hormones[1], environment[2], carbohydrates[3], C/N ratio[4], light, temperature[5,6] and so on. Among these factors, more and more studies show that hormones, either alone or in combination, plays a very important role in bolting[1]. Our team previously identified two cultivars, S24 and S39, through many years of field trials. S39 is prone to bolting and is very sensitive to high temperature, whereas S24 is bolting resistant and heat insensitive. RNA-sequencing (RNA-Seq) data showed that 12,204 genes were differentially expressed in S39 vs. S24; 8 genes associated with gibberellin (GA) were found, and exogenous gibberellin3(GA3) significantly promoted bolting in the two cultivars[7]. GAs regulate various plant growth and developmental pathways, such as stem elongation and flower development by promoting cell division and cell elongation[8]. Research by Sun et al. showed that GA treatment specific concentrations can significantly shorten the cabbage flowering time while regulating stem length and the bolting period[9]. In addition, comprehensive analysis has shown that GA plays an important role in the regulation of bolting in lettuce, though the specific regulatory mechanism remains unclear. Similar to exogenous GA can promote bolting [7], IAA has different functions in different bolting varieties [10,11]. Besides, Song's study speculated that ABA functions in a manner similar to that of GA4 in early bolting [12], so we guessed that ABA also has the same effects in lettuce. For the hormones are important during bolting, the hormones changes that occur prior to bolting are worth noting.

To preliminary explore the molecular mechanism and regulation of bolting and to detect changes in lettuce during bolting promoted by high temperature, stems and leaves of the bolting-sensitive cultivar S39 grown at room and high temperatures were selected for transcriptome sequencing. Morphological changes, changes in endogenous hormones in the stems and leaves of S39 plants treated with high and normal temperatures during bolting and analyzed the effects of hormone levels on the process., and stem and leaf transcriptome data showed that GA and certain transcription factors play an important role in regulating bolting in lettuce. These findings provide a theoretical basis for further study on bolting in lettuce.

## Materials and methods

### Plant materials and growth conditions

The heat-sensitive *Lactuca sativa* L. cultivar S39 was obtained from College of Plant Science and Technology, Beijing University of Agriculture. Seeds were germinated in cell trays filled with soil in a growth chamber. When the seedlings had five leaves, 60 plants were randomly divided into two groups to be planted in plastic cups. After one week, the seedlings were treated at different temperatures: 37°C or 25°C as a control. After treatment, the seedlings were monitored for 0–9 d.

### Paraffin sectioning

Flower buds were washed with distilled water and fixed using glutaraldehyde fixative before being stored in a refrigerator at 4°C. We selected a sarranine and fast green staining method to observe cytological changes. The samples were fixed, subjected to 7 levels of dehydration and xylene, and embedded. Sections were then sliced, dewaxed, and stained with sarranine and fast green before sealing. The slice thickness was 10 μm. The slices were observed under a microscope and photographed.

### Exogenous hormone application

There were four different treatment groups which were all cultivated in room temperature(25°C) in order to control a single variable: 50 mg/L GA3 and 200 ppm chlormequat chloride (CCC) which were chosen as positive control, water which was used as the negative control, and untreated plants which was used as blank control. Plants at the fifth-true leaf stage with uniform growth were selected. Twelve plants were used for each treatment, and the stem length was measured every twice daily for 12 days after treatment.

### Determination of endogenous hormone contents

For quantitative analysis of hormone contents, 0.1 g of lyophilized stems and 0.2 g of leaves were homogenized in methanol, and the contents were quantified by competition enzyme-linked immunosorbent assay (ELISA). Three plants were randomly selected for sampling, and the fifth leaves were used to determine the endogenous hormone levels. Student *t*-test was used to test whether S39 of control and treatment group showed significant difference in concentration of each endogenous hormone using *p* < 0.05 as significance cutoff.

### RNA extraction and quality assessment and quantitative reverse-transcription polymerase chain reaction (RT-qPCR)

Total RNA was isolated from each sample using an ultra pure total RNA rapid extraction kit (Aidlab). Only RNA samples that passed quality assessment were chosen for RNA-Seq analyses. cDNAs were reverse-transcribed from 2 μg total RNA using the TransScript First-Strand cDNA Synthesis SuperMix (Aidlab), and RT-qPCR was performed with an ABI PRISM 7500 Real-Time PCR System (Applied Biosystems, USA). The lettuce 18S ribosomal RNA (GeneBank number: HM047292.1) was used as an reference gene. Each RT-qPCR experiment was performed with three biological replicates and technical replicates (3 × 3). The relative expression of each gene was calculated using the 2 ^-ΔΔc(t)^ method[13] and standard deviation was calculated among three biological replicates.

### Identification of differentially expressed genes (DEGs)

After low-quality regions and adaptors were trimmed through pre-processing, reads were aligned to the lettuce genome (http://lgr.genomecenter.ucdavis.edu/Home.php) using HISAT2^4^. The number of reads for each gene was obtained using the Python package HTSeq-Count[14], and the identification of differentially expressed genes was performed with the R package edgeR[15]. Expression of each gene was normalized to CPM (counts per million), and genes with a CPM value greater than 1 in at least 3 samples were retained for further analysis. In one comparison, genes were regarded as differentially expressed when their expression was at least 2-fold altered compared to the other and the false discovery rate (FDR) was less than 0.05.

### Gene ontology (GO) enrichment analysis

GO enrichment analysis of up- and down-regulated DEGs was performed using the R package TopGO[16], which assesses the significance of GO enrichment using Adrian Alexi’s improved weighted scoring algorithm and Fisher’s exact test. GO terms with a p-value less than 0.05 were considered to be significantly enriched.

### MapMan functional analysis

MapMan was used to elucidate the functions of DEGs. First, Mercator online software [17] was used to annotate the lettuce genome and obtain the mapping file that MapMan [18] requires. The mapping file together with the DEG foldchange file were the input files for MapMan and used for functional categorization analysis.

## Results

### Morphological changes in lettuce due to differential temperature treatment

**Fig 1:**
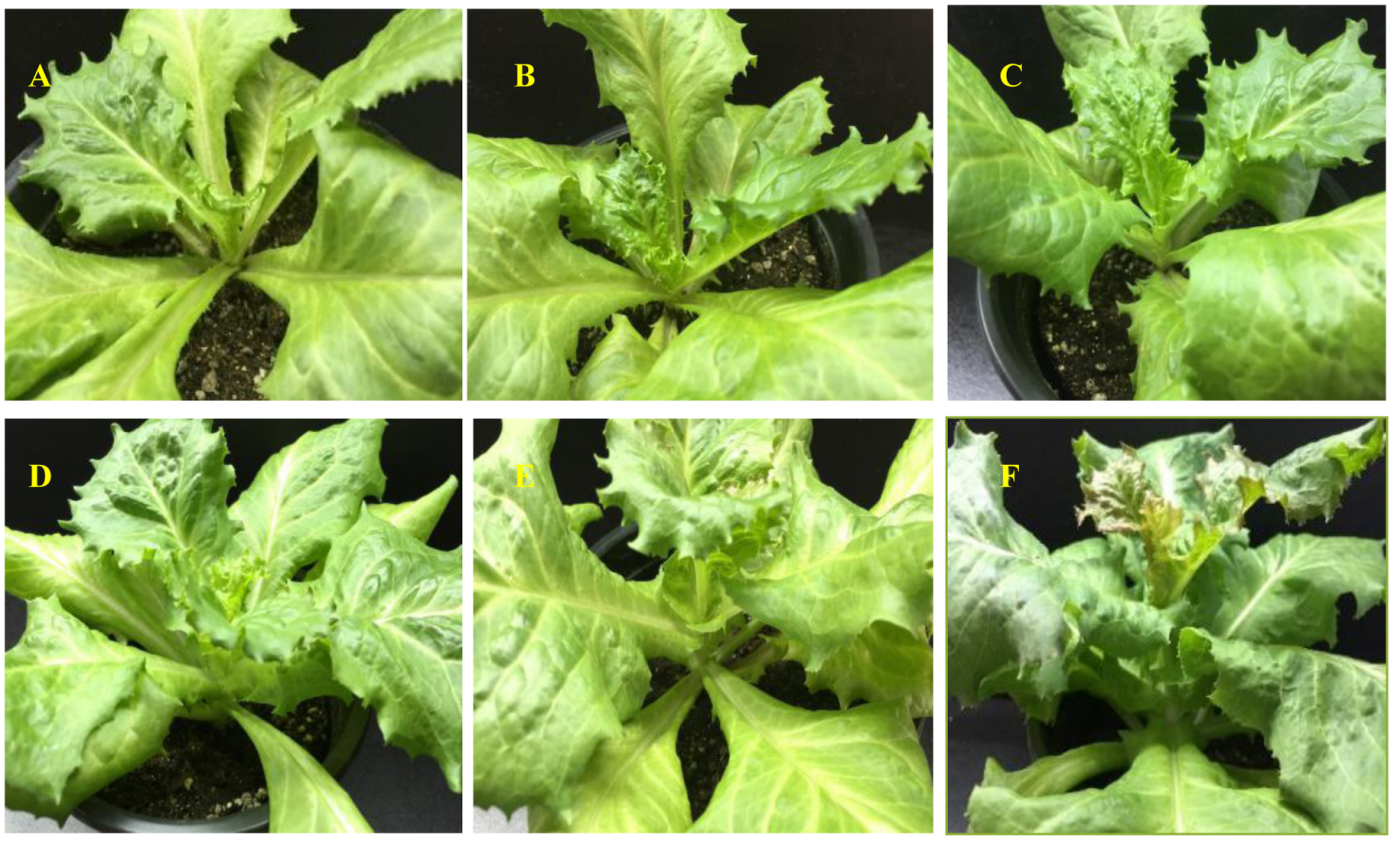
Comparison of visual morphology between high-temperature(37°C) and control groups(25°C). (A-C) Control plants on days 5, 7, and 9, respectively. (D-F) High-temperature treatment plants on days 5, 7, and 9, respectively.

To examine the metabolic mechanisms of S39 plants under high temperature, a control group was cultured at control temperature (25°C), and another group was treated with a simulated summer temperature (37°C). By comparing morphological changes, we found no obvious differences between the high- (Fig 1A), and control-temperature groups until the fifth day (Fig 1D). The treatment group began bolting on the seventh day (Fig 1E), and changes in the stem were obvious on the ninth day (Fig 1F), as these plants were significantly taller than those in the control group (Fig 1C). Bolting did not occur in the control group during the treatment period(Fig 1A and 1C).

**Fig 2:**
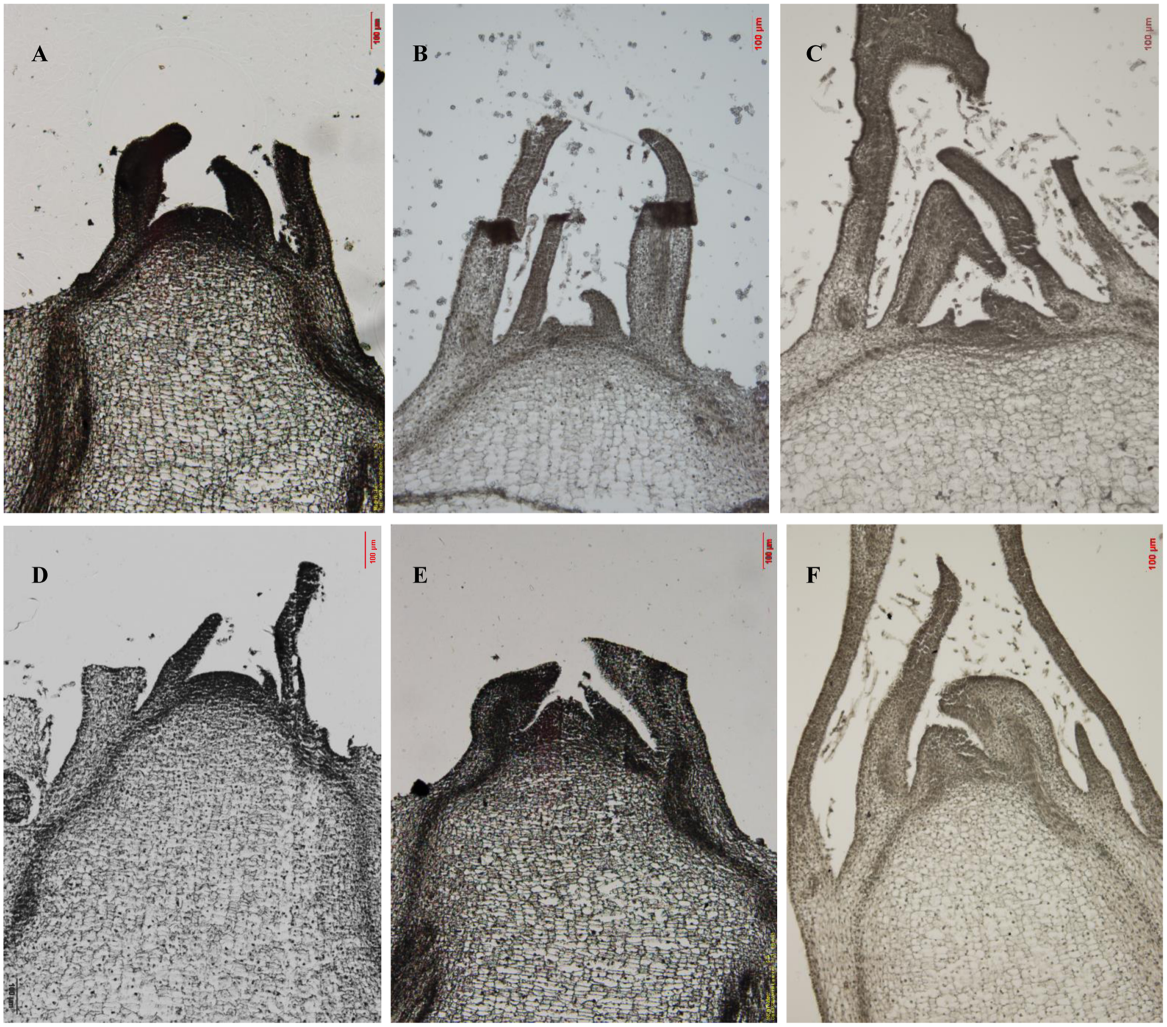
Comparison of paraffin sections from different treatments. (A-C) Control shoot tip meristems on days 5, 7, and 9, respectively, which did not differentiate over time. (D-F) Treatment shoot tip meristems on days 5, 7, and 9, respectively, with gradual growth observed.

Paraffin sections were also used to observe changes in shoot tip growth. On the fifth day of treatment, the tip of the high-temperature group had a flat and smooth surface with young leaves and leaf primordia (Fig 2A). However, differences between the treatment and control groups were not obvious. On the seventh day, the growing cone had increased in size and was significantly longer in the high-temperature group than in the control group (Fig 2B). The growing cone increased in size until the ninth day (Fig 2C). Conversely, the control group growing point did not change in size during the treatment period.

### Changes in endogenous hormone contents before and after bolting

**Fig 3:**
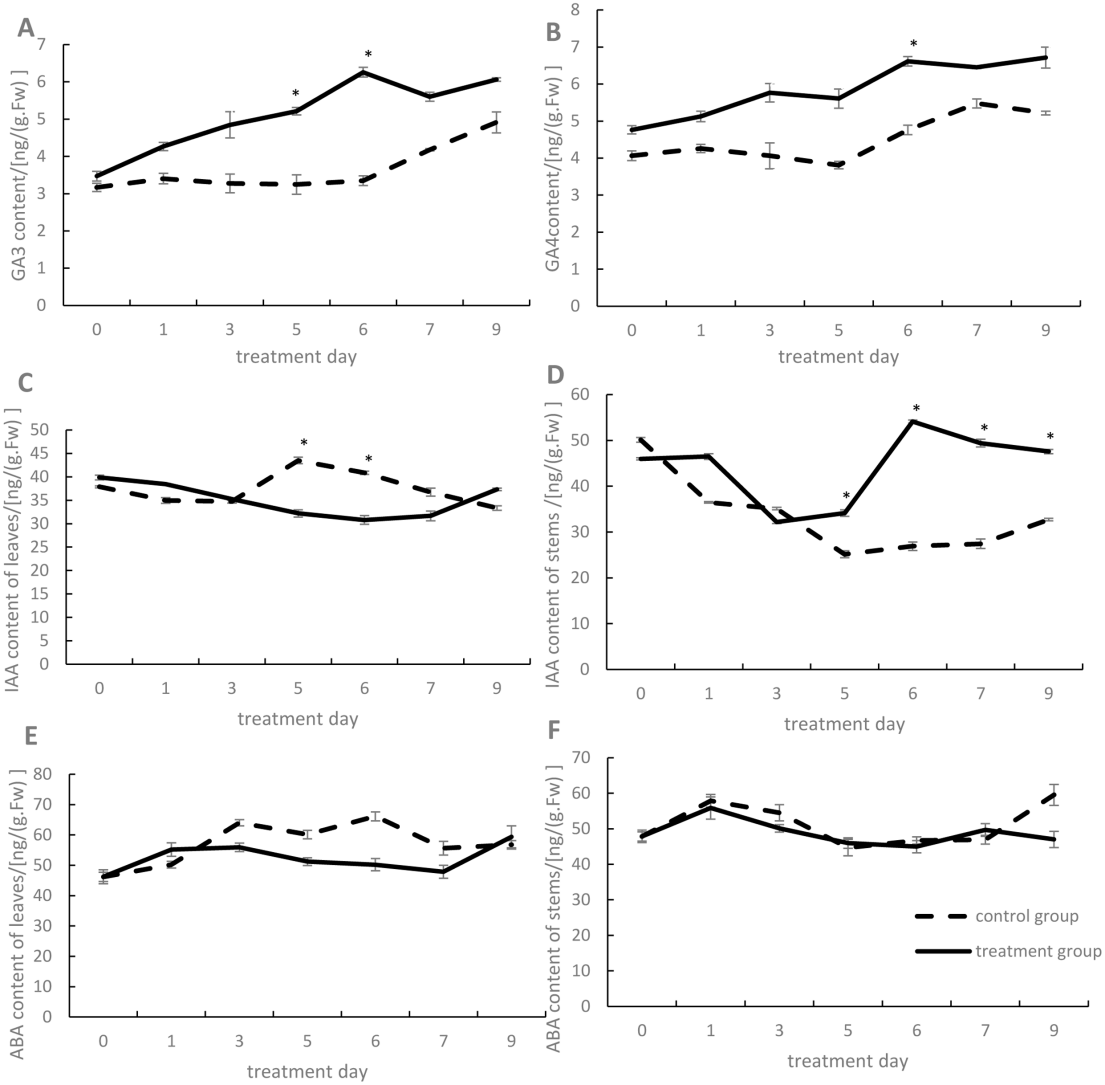
Hormone contents in different parts of lettuce plants on different treatment days. (A and B) GA3 and GA4 contents in leaves, respectively. (C and D) IAA contents in leaves and stems exposed to different treatments, respectively. (E and F)ABA contents in leaves and stems exposed to different treatments, respectively. The solid line represents the changes of treatment group, and the dotted line represents the control group. Asterisks represent significant difference at *P* < 0.05 by student *t*-test.

We found that the GA3 (Fig 3A) and GA4 (Fig 3B) contents in leaves reached a maximum on the sixth day after high temperature treatment, significantly higher than the control group and reach 187% and 138% of the control group, respectively, and these high levels were maintained. In contrast, there were no obvious changes in GA3 and GA4 levels in stems.

The stem IAA contents reached a maximum on the sixth day (Fig 3D) and significantly higher than the control group, and it reach 201% of the control, then maintained the high IAA levels in the following days. However, after high-temperature treatment, IAA levels showed a decreasing trend in leaves on the sixth day (Fig 3C). No notable changes in ABA contents in stems and leaves were observed (Fig 3E and 3F).

### Stem and leaf transcriptome analyses of S39 plants by RNA-Seq

The bolting time in S39 plants has been determined. To explore the genes and gene networks involved in regulating bolting, stem tips and leaves from plants treated at room and high temperatures were selected as materials for transcriptome sequencing on the seventh day after treatment. Three biological replicates were performed for each treatment. Using a fold change ≥2 and an FDR <0.05 as cutoffs, 1216 and 933 genes (S1 and S2 Figs) were found to be differentially up-regulated and down-regulated, respectively, in the stem of the treatment group versus the control group; 1443 and 1038 genes(S1 and S2 Figs) were differentially up-regulated and down-regulated in leaves (S1, S2, S3 and S4 Tables)

### Validation of RNA-Seq data by RT-qPCR

**Fig 4:**
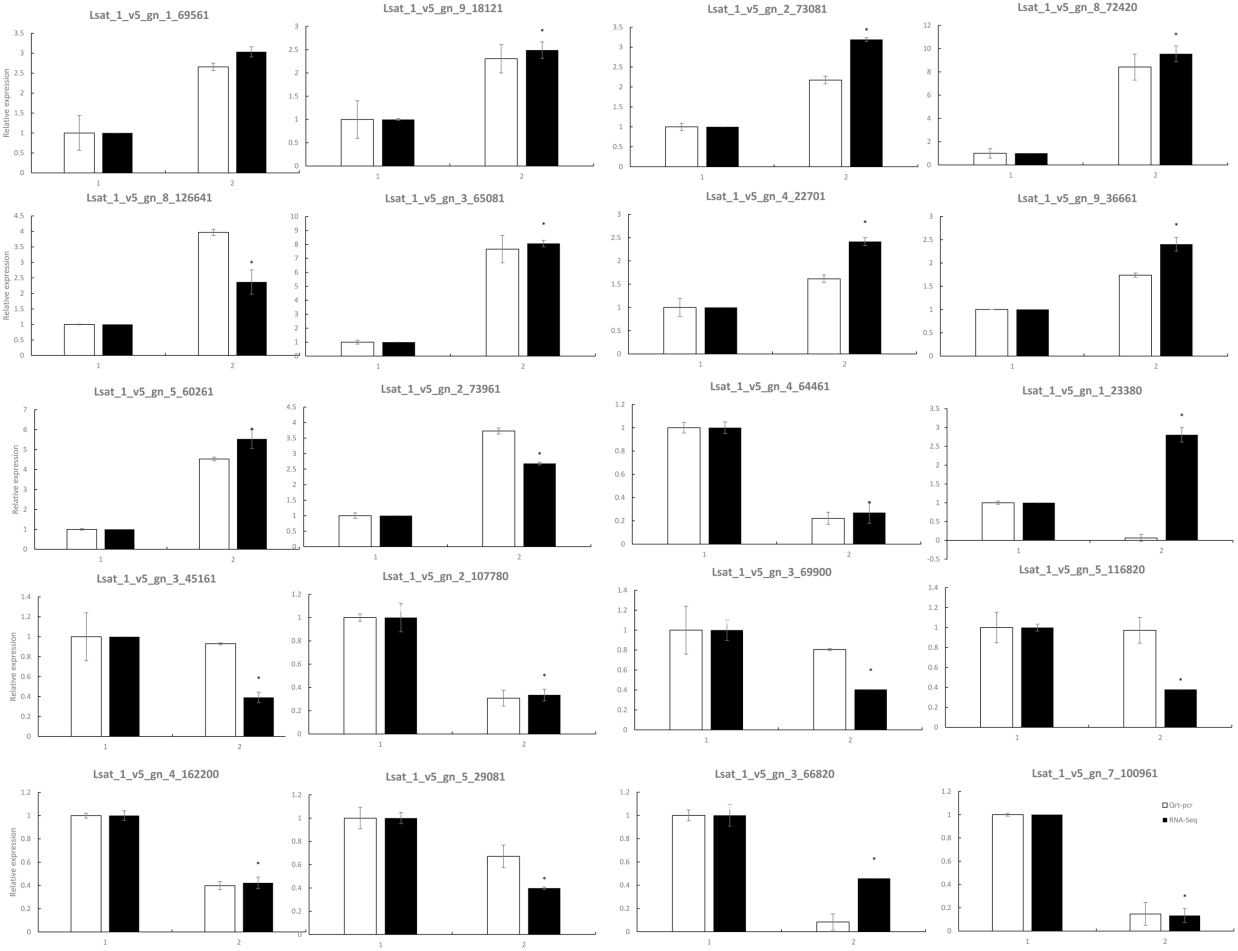
Verification of differentially expressed genes by RT-qPCR. Twenty genes were chosen for RT-qPCR validation. The white and black bars represent the relative expression levels of each gene in the control and high-temperature groups, as detected by RT-qPCR and RNA-Seq, respectively. To plot the RNA-Seq data, gene expression in the control group was set to be the same as that observed by RT-qPCR, and relative expression in the high-temperature group was calculated using the fold-change detected by RNA-Seq. The bars represent the standard deviation (n = 3); 1 represents the control temperature, and 2 represents the high temperature. Asterisks indicate that the gene transcriptions are significantly different between control and treatment group (unpaired t test, P< 0.05).

To verify the DEGs identified by RNA-Seq, we performed RT-qPCR assays using independently collected samples that were in the same developmental stage of the stems and leaves used for the RNA-Seq analysis. Among the 20 randomly selected DEGs from stem tips and leaves, 11 genes showed higher expression and 9 lower expression in S39 plants (Fig 4), 19 out of the 20 genes showed the same changes patterns in the RT-qPCR assays as in the RNA-Seq data. The pearson correlation coefficient between RT-qPCR and RNA-Seq data was 0.934, indicating that the RNA-Seq data were highly reliable.

### Functional analyses of DEGs

**Fig 5:**
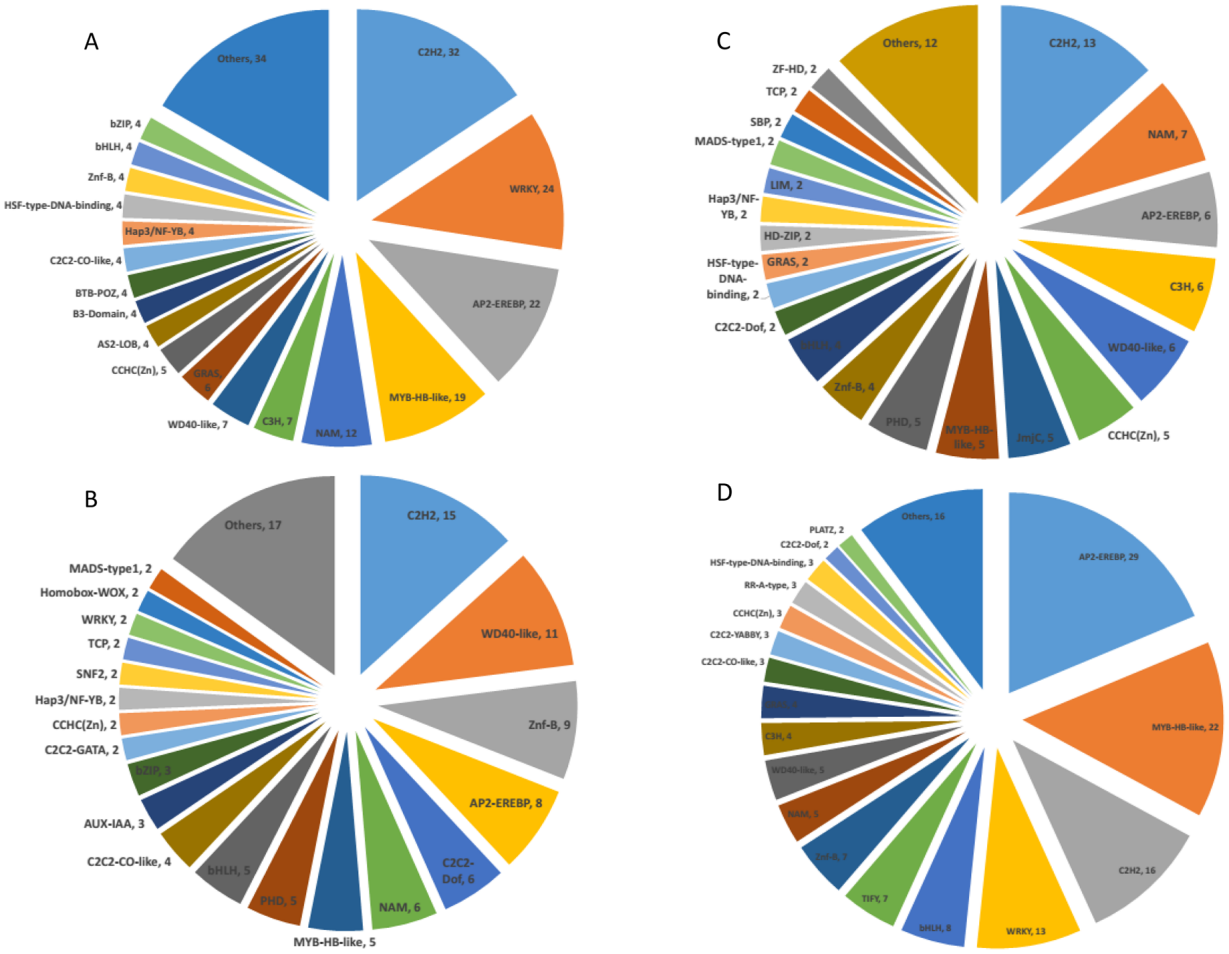
Gene Ontology (GO) analysis of down- and up-regulated genes in the high-temperature group vs. the control group. GO terms belonging to biological process (BP), molecular function (MF), and cellular component (CC) groups are shown in red, green, and blue, respectively. (A and B) Up- and down-regulated genes in the stem tip, respectively. (C and D) Up- and down-regulated genes in leaves, respectively. GO terms are sorted based on their *p*-values.

GO annotation was used to identify changes in biological process (BP) and molecular function (MF) between stem tips and leaves. In the stem tip, among the genes that were down-regulated in the treatment group, the top four significantly enriched GO terms in the BP category were “photosynthesis, light harvesting”, “lipid metabolic process”, “metabolic process” and “regulation of transcription, DNA-templated” (Fig 5A). The top four significantly enriched GO terms in the BP category in leaves were “photosynthesis, light harvesting”, “oxidation-reduction process”, “glycolytic process” and “sodium ion transcription” (Fig 5C). These results suggest that expression of photosynthesis-related genes was significantly decreased after heat stress. For example, *Lsat_1_v5_gn_1_95341*, a gene that encodes chlorophyll A/B binding protein 1, was down-regulated by 11.1-fold (Log_2_FC = -3.47) and 10.9-fold (Log_2_FC = -3.44) in the stem and leaf, respectively.

Among up-regulated DEGs, “defense response” and “signal transduction” were significantly enriched for BP in the stem tip (Fig 5B); “regulation of transcription, DNA-templated” and “defense response” were significantly enriched in the leaf (Fig 5D). These results show that plant responses were enhanced after high-temperature treatment.

Han found 7 genes associated with GA that can regulates bolting in lettuce [7]; therefore, we focused on changes in GA-related genes at the transcriptional level (Table 1). Three GA-related genes were detected in the stem tip, 2 of which were down-regulated and 1 of which was up-regulated. In contrast, only 3 up-regulated genes were observed in leaves. However, it is interesting that the same genes showed opposite trends when expressed at different sites; for example, *Lsat_1_v5_gn_7_100961* was down-regulated by 7.5-fold (Log_2_FC = -2.90) in the stem and up-regulated by 5.6-fold (Log_2_FC = -2.48) in leaves. RT-qPCR revealed a 7-fold up-regulation and a 4.9-fold down-regulation of this gene in the stem and leaves, respectively. These results are similar to the transcriptome results, highlighting the consistent changes in endogenous GA3 in stems and leaves after high-temperature treatment. In Han’s study, among the 7 genes associated with GA, 4 are homologous to *Arabidopsis thaliana* AT1G74670.1 and 1 to *A*. *thaliana* AT5G59845.1. However, in this study, we found 6 genes associated with GA, 2 of which are homologous to AT1G74670.1 and 2 to AT5G59845.1. The findings are consistent with Han’s study, demonstrating that gibberellin-regulated genes are involved in bolting in lettuce.

**Table 1:** List of GA-related genes that were differentially expressed in the lettuce stem and leaf in the high-temperature group vs. the control group.

location	GeneID	Putative function	Arabidopsis homolog	Log_2_FC	FDR
stem	Lsat_1_v5_gn_7_100961	gibberellin 2-oxidase	AT1G30040.1	-2.90788	5.27E-16
stem	Lsat_1_v5_gn_4_162200	Gibberellin-regulated family protein	AT5G59845.1	-1.24419	4.88E-21
stem	Lsat_1_v5_gn_1_23380	Gibberellin-regulated family protein	AT5G59845.1	1.4888	1.16E-08
leaf	Lsat_1_v5_gn_7_100961	gibberellin 2-oxidase	AT1G30040.1	2.47716	2.74E-06
leaf	Lsat_1_v5_gn_9_31921	Gibberellin-regulated family protein	AT1G74670.1	1.243024	0.034798
leaf	Lsat_1_v5_gn_2_90361	Gibberellin-regulated family protein	AT1G74670.1	1.177208	0.000335

### TFs are implicated in lettuce bolting

**Fig 6:**
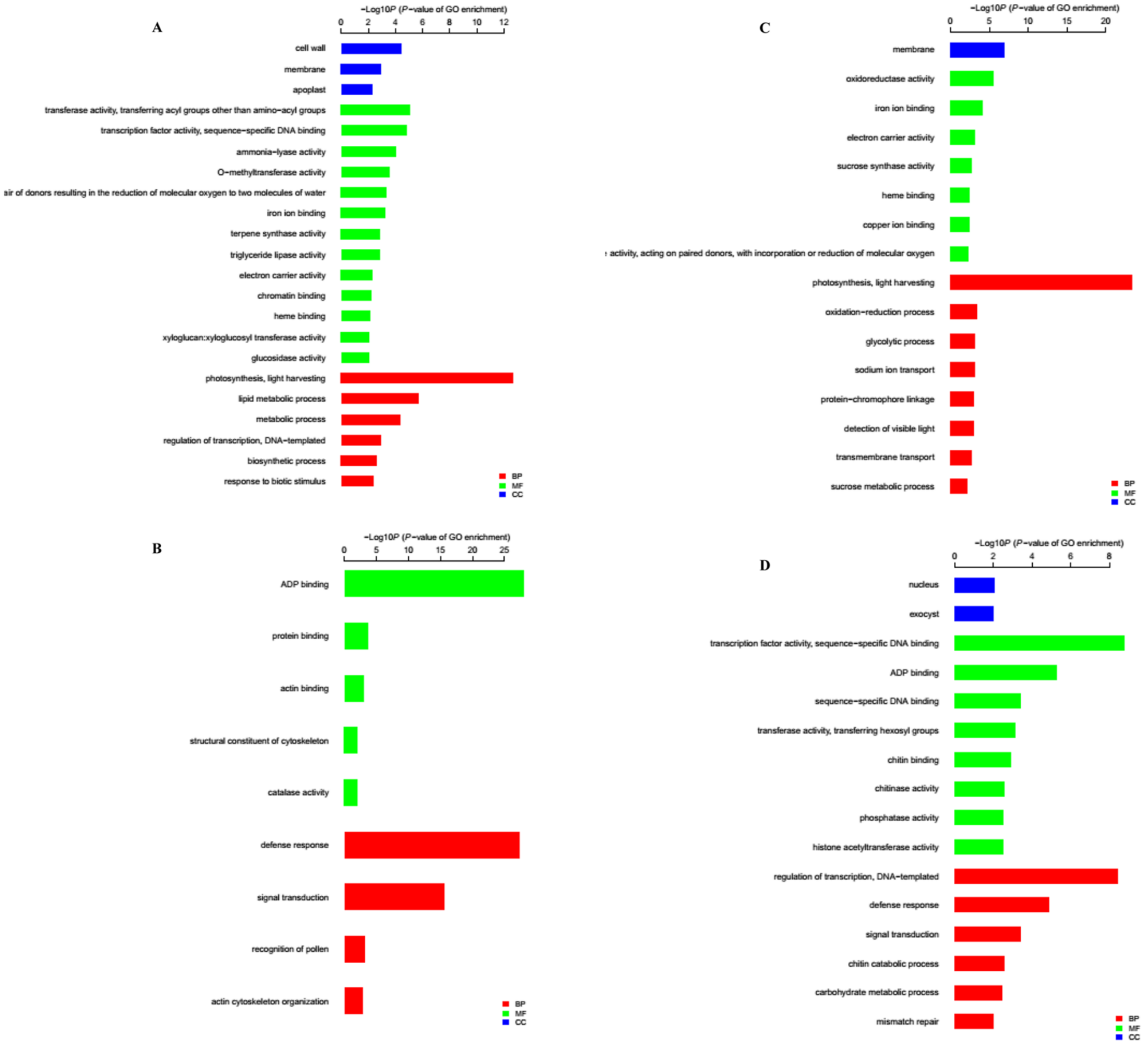
Family assignment of transcription factors that showed differential expression in the high-temperature group vs. the control group. The number of genes assigned to each family is shown behind a comma. (A and C) Genes with higher expression and lower expression in the stem tip, respectively. (B and D) Genes with higher expression and lower expression in leaves, respectively.

Analysis of transcriptome data indicated that lettuce contains a large number of TFs, the complexity and importance of which have been shown in various species [19]. Thus, we next analyzed major TFs. Among up-regulated TFs, 13, 7 and 6 genes were assigned to the C2H2, NAM, and AP2-EREBP families, respectively, in the stem tip (Fig 6C) and 32, 24, and 22 to the C2H2, WRKY and AP2-EREBP families, respectively, in the leaf (Fig 6A).

WRKY TFs, which are widespread and comprise a relatively large family in plants, are mainly involved in plant stress responses [20,21], regulating growth and development. In our study, WRKY TFs were only enriched in leaves; for example, LsWRKY (*Lsat_1_v5_gn_6_12161*) showed a 17.7-fold reduction when detected by RNA-Seq and a 19.5-fold decrease when detected by RT-qPCR (Fig 4). Twenty-four WRKY TFs showed an average decrease in expression of 4.9-fold.

C2H2 zinc finger protein family members are mainly involved in regulating the development of floral organs, seeds and seedlings and in modulating the expression of genes related to stress resistance[22]. In the leaf, the C2H2 TF *Lsat_1_v5_gn_8_16081* showed a 67.4-fold increase in expression. Overall, C2H2 TFs were enriched in different plant parts, with various expression patterns, suggesting that the C2H2 family has important connections to bolting in lettuce.

Moreover, TFs from other families, such as LsAP2 (*Lsat_1_v5_gn_2_88660*) and LsMADS (*Lsat_1_v5_gn_4_71361*), showed 8.42-fold and 3.97-fold higher expression, respectively, in the stem tip. In leaves, LsAP2 (*Lsat_1_v5_gn_9_53960)* and LsMYB (*Lsat_1_v5_gn_9_24661*) showed 510.2-fold and 10.86-fold higher expression, respectively.

We next analyzed down-regulated TFs, of which we detected 156 (Fig 6D) and 113 (Fig 6B) in stem tips and leaves, respectively, in the S39 plants. In the stem tip, 29, 22 and 16 genes in the AP2-EREBP, MYB-HB-like, and C2H2 families, respectively, were found; in leaves, 15, 11 and 9 genes in the C2H2, WD40-like, and Znf-B families, respectively, displayed reduced expression. We also observed an interesting phenomenon whereby the same TFs showed opposing trends between stems and leaves. For example, *Lsat_1_v5_gn_6_92900*, an AP2-EREBP family TF, was down-regulated by 36.8-fold (l0g_2_FC = -5.2) in the stem but up-regulated by 25.1-fold in leaves (l0g_2_FC = 4.65); these changes were similar to those of endogenous GA3, which may ensure plant homeostasis.

### Exogenous spraying of GA3 and CCC

According to transcriptome data sets, GA contents in S39 plants were significantly increased after temperature treatment. To investigate the effects of GA on S39 plants, we treated plants with the exogenous plant growth regulator GA3[23] and its biosynthetic inhibitor CCC[24]in the same amount of water or treated controls plants with water in room temperature(25°C). In total, there were four different treatment modes applied at the fifth-leaf stage. We observed changes in stem length and endogenous hormone contents in leaves and stems after different treatments at the control temperature (25°C). Three biological replicates were performed for each treatment, and the results showed significant changes in S39 plants under the different treatments.

**Fig 7:**
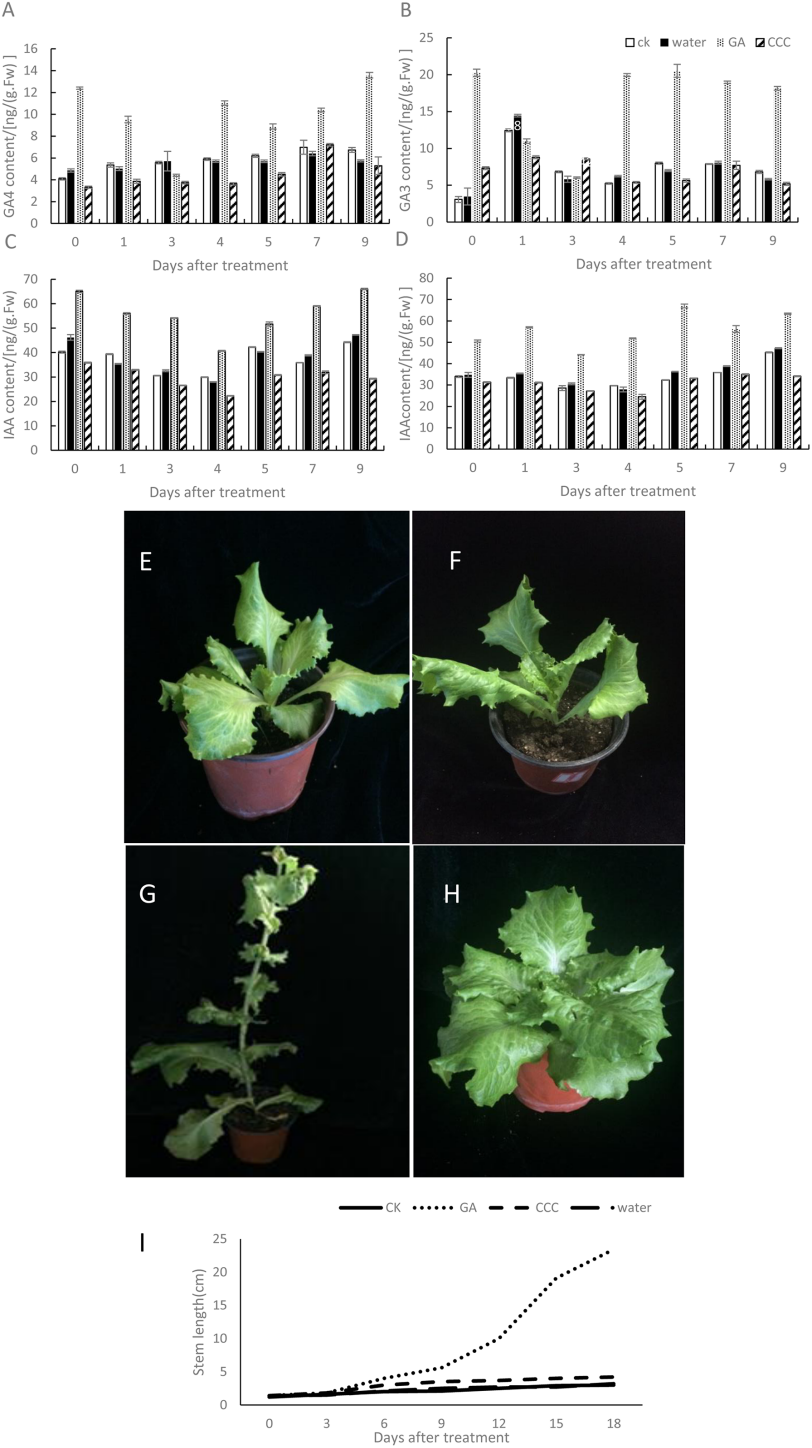
Changes in hormone contents, morphology and stem length after treatment with exogenous hormones. Plants in the fifth-leaf stage were treated with the exogenous plant growth regulator GA3 or its biosynthetic inhibitor CCC in the same amount of water. There were four different treatment modes. Changes in stem length and endogenous hormone levels in leaves and stems were examined at the control temperature after the different treatments. (A and B) GA4 and GA3 contents in leaves. (C and D) IAA contents in leaves and stems. (E-H) The morphology of lettuce plants without any treatment or treated with water, GA or CCC, respectively. (I) Stem elongation in S39 plants after treatment with exogenous hormone for 18 days.

Our data showed that bolting begins in the fifth day after GA3 treatment in S39 plants. The stem length increased significantly (Fig 7I), as did the blade spacing, with the lower blade being larger than the upper (Fig 7G). GA3 (Fig 7B) and GA4 (Fig 7A) levels in leaves first fell and then rose; the contents of both GAs increased significantly by the fourth day and were maintained at high levels. GA3 and GA4 in the stem exhibited a decreasing trend, which was consistent with the results after high-temperature treatment. The findings further confirmed that high GA3 and GA4 levels in leaves play a key role in bolting. In stems and leaves, the endogenous hormones showed opposite trends to maintain a dynamic balance. In addition, changes in IAA contents showed different trends after the different treatments. The IAA content in the stem was the highest on the fifth day, 184% higher than in the control group, and a high IAA content was maintained after the fifth day (Fig 7D). The IAA content in leaves showed a decreasing trend (Fig 7C), a change that was consistent with the results after high-temperature treatment.

After CCC treatment, S39 plants became compact and stout with dense large leaves; bolting did not occur during this period. GA3 and GA4 contents were reduced in stems and leaves; similarly, IAA levels also decreased in stems and leaves. The ABA contents in stems and leaves were not significantly altered after treatment with either GA or CCC. The water-treated and untreated plants did not bolt during the treatment period, and there were no significant changes in GA3, GA4, IAA and ABA contents in the controls.

## Discussion

### Determination of the bolting period after high-temperature treatment

Bolting signifies the transition from vegetative to reproductive growth in lettuce and is a key stage of the plant life cycle[7]; thus, determining the bolting period of a given plant is important for future research. After high-temperature treatment, S39 plants began bolting on the seventh day (Fig 1E). Paraffin sections also showed that the growing cone increased significantly in size compared to the control group on the seventh day (Fig 2B). The increase observed during the treatment period continued for both the stem length and growing point. Therefore, we determined that S39 began to bolt after seven days.

### Analysis of endogenous hormone contents under different treatments

After high-temperature treatment, the GA3 and GA4 contents in leaves increased and reached a maximum on the sixth day, with values 187% and 138% higher, respectively, significantly higher than the control group. These hormone contents remained high thereafter (Fig 3A and 3B), suggesting that high GA levels in leaves promote bolting and increase stem length. However changes in GA3 and GA4 levels in stems were not obvious and even displayed opposite trends compared to leaves; this may be due to the dynamic balance of GA3 and GA4 between leaves and stems. Moreover, IAA contents in stems increased significantly by the fifth day, up to 135% higher than in the control group, and reached a maximum on the sixth day, which was 201% significantly higher than in the control group. IAA levels in stems also remained high (Fig 3D), suggesting that high IAA levels in the stem promote bolting. In contrast, the leaf IAA content showed a decreasing trend (Fig 3C), likely due to the transport of IAA from leaves to the stem through active and polar transport. ABA levels did not significantly change in stems or leaves during the treatment period, suggesting that there is no correlation between ABA and bolting in lettuce(Fig 3E and 3F).

After spraying with GA3, the lettuce plants began bolting on the fifth day; GA3 and GA4 levels in leaves initially decreased and then increased, after which they remained high (Fig 7A and 7B). The observed decrease occurred because exogenous GA3 was not rapidly absorbed; thus, the residual GA3 content in leaves gradually decreased. After exogenous GA3 was absorbed by the plant, the GA3 and GA4 contents increased gradually and promoted bolting. GA3 and GA4 levels remained high thereafter, with the same results as found with the high-temperature treatment. Spraying the lettuce plants with CCC did not promote bolting and inhibited GA3 and GA4 synthesis. The water only- and untreated plants did not display bolting, and the GA3 and GA4 contents in these samples showed no significant changes. Therefore, we believe that high GA3 and GA4 levels in leaves promote bolting. The highest content of IAA was found on the fifth day, with a high content being maintained in stems (Fig 7D). This change is consistent with the results of high-temperature treatment; thus, we believe that a high IAA content in the stem promotes bolting. After CCC treatment, IAA levels in stems and leaves were lower than in the control group, showing that CCC reduced the IAA content and subsequently inhibited bolting. There were no significant changes in the ABA content in stems or leaves after GA or CCC treatment, which confirmed that ABA does not significantly affect bolting in lettuce.

### Analysis of transcription factors involved in bolting

Genome sequence analysis of the fourth *Arabidopsis* chromosome revealed that 15% of genes encode or may encode TFs[25]. There are a large number of TFs, and their complexity and importance in regulation have been demonstrated in various species [26]. Moreover, a previous study showed that TFs plays an important role in bolting[27,28] and that different TFs have different functions throughout the plant life cycle [29,30].

Similar to cucumber[31], TFs in different parts of the plant exhibit varied enrichment. Data obtained thus far demonstrate that different TFs play different roles in plant growth and development[32]. As the complexity and importance of regulation by TFs have been confirmed in many species [19], a large number of TFs may have be involved in bolting. We searched for TFs in stems and leaves that displayed differential expression and thus may play key roles in regulating bolting in lettuce. In the stem, a total of 258 TFs were identified, of which 98 and 156 were differentially up- and down-regulated, respectively, in the treatment group compared to the control group; in leaves, 202 and 115 TFs were differentially up- and down-regulated, respectively. RT-qPCR confirmed that our RNA-Seq data were highly reliable. The observed stem and leaf TFs are largely members of the C2H2 zinc finger and AP2/EREBP families. The same TFs families were found to be enriched at different levels, suggesting that TFs play important roles in regulating bolting. In stems, 13 and 6 C2H2 and AP2 family TFs, respectively, were up-regulated; 16 and 29 C2H2 and AP2 family TFs, respectively, were down-regulated. In leaves, 32 and 22 C2H2 and AP2 family TFs, respectively, were up-regulated; 15 and 18 C2H2 and AP2 family TFs, respectively, were down-regulated.

C2H2 zinc finger family TFs are primarily related to plant growth and development as well as the response to environmental stress [33]. A previous study showed that the *Arabidopsis* SUPERMAN zinc finger protein plays an important role in flower development[34,35]. This TF family can also control growth and development by regulating plant hormone contents. For example, AtGIS, a C2H2 zinc-finger transcription factor from *Arabidopsis*, regulates glandular trichome development through GA signaling in tobacco [36]. In the present study, the C2H2 family TF LsC2H2 (*Lsat_1_v5_gn_4_139200*) showed 24.9-fold lower expression in the stem, and *Lsat_1_v5_gn_8_16081* showed 67.4-fold higher expression in leaves; this high difference ratio suggests that C2H2 family TFs are enriched in different parts of the plant and display differential expression. Thus, we believe that the C2H2 family may have important links to bolting in lettuce. There are many C2H2 family TFs, and each gene has a different role in regulation. Moreover, different genes in the same family may exhibit different trends in stems and leaves, resulting opposite regulation. For example, *Lsat_1_v5_gn_6_78761* showed 7.07-fold (log_2_FC = -2.82) lower expression in the stem but 12.57-fold (log_2_FC = 3.65) higher expression in leaves. Similarly, AP2/EREBP family TFs, which are involved in endogenous hormone signaling, regulate expression of downstream genes and enhance resistance and tolerance, also displayed similar trends. *Lsat_1_v5_gn_6_92900*, an AP2-EREBP family TF, was down-regulated by 36.8-fold (l0g_2_FC = -5.2) in the stem but up-regulated by 25.1-fold (l0g_2_FC = 4.65) in leaves. AP2/EREBP was both up- and down-regulated by treatment, with down-regulation occurring in the stem and significant up-regulation in leaves, indicating that AP2/EREBP may be involved in responses to various plant hormones that elicit a stress response. Because many TFs display opposing trends in stems and leaves, we suspect that endogenous hormones are closely related to these TFs.

When lettuce plants are treated with high temperature, the leaves are the first organ to experience the temperature change and generate a stress response [37]. Higher WRKY expression in leaves is related to many physiological processes [38], including disease and damage resistance, growth regulation, and development. Expression of LsWRKY (*Lsat_1_v5_gn_6_12161*) was 19.5-fold higher under high temperature, and 24 WRKY TFs were increased by an average of 4.9-fold, suggesting that WRKY TFs can help lettuce increase resistance and tolerance capacities after high-temperature stress while also promoting normal growth under heat stress conditions.[39] WRKY TFs also regulate genes that are involved in hormone interactions [40,41], thus, the impact of highly enriched WRKY TFs on hormone changes is worth exploring. Additionally, many TFs displayed changes in expression after heat stress; for example, LsWD40 (*Lsat_1_v5_gn_8_108400*), which is involved in meristem formation, seedling development and flower development, showed 65.9-fold higher expression in the stem tip. LsMYB (*Lsat_1_v5_gn_1_19840*), a gene related to embryo development [42], generative cell division and differentiation and stem morphogenesis, was down-regulated by 24.9-fold in the stem. Therefore, we suspect that these TFs play important roles in bolting. We speculate that changes in TFs regulate the expression of genes that further regulate endogenous hormones, thereby promoting lettuce bolting. The function and regulatory mechanism is worthy of further study, and our results lay a foundation for molecular breeding in lettuce to enable planting at different times of the year.

## Supporting information

S1 Fig Venn diagram of down-regulated genes in the high-temperature group vs. the control group. GY and CY represented leaves of high-temperature group and control group. GJ and CJ represented the stem tip of high temperature group and control group, respectively.
(PDF)

S2 Fig Venn diagram of up-regulated genes in the high-temperature group vs. the control group. GY and CY represented leaves of high-temperature group and control group. GJ and CJ represented the stem tip of high temperature group and control group, respectively.
(PDF)

S1 Table List of genes that were significantly down-regulated in high temperature group and control group in stem tip. (XLS)

S2 Table List of genes that were significantly up-regulated in high temperature group and control group in stem tip. (XLS)

S3 Table List of genes that were significantly down-regulated in high temperature group and control group in leaves. (XLS)

S4 Table List of genes that were significantly up-regulated in high temperature group and control group in leaves. (XLS)

S5 Table List of RT-qPCR primer sequences. (XLSX)
