# Correction: Transcriptomic analysis reveals the roles of gibberellin-regulated genes and transcription factors in regulating bolting in lettuce (*Lactuca sativa* L.)

**DOI:** 10.1371/journal.pone.0221609

**Published:** 2019-08-28

**Authors:** Xueying Liu, Shanshan Lv, Ran Liu, Shuangxi Fan, Chaojie Liu, Renyi Liu, Yingyan Han

The gene LsWRKY (Lsat_1_v5_gn_6_12161) incorrectly did not appear in Fig 4. Please see the corrected Fig 4 here.

**Fig 4:**
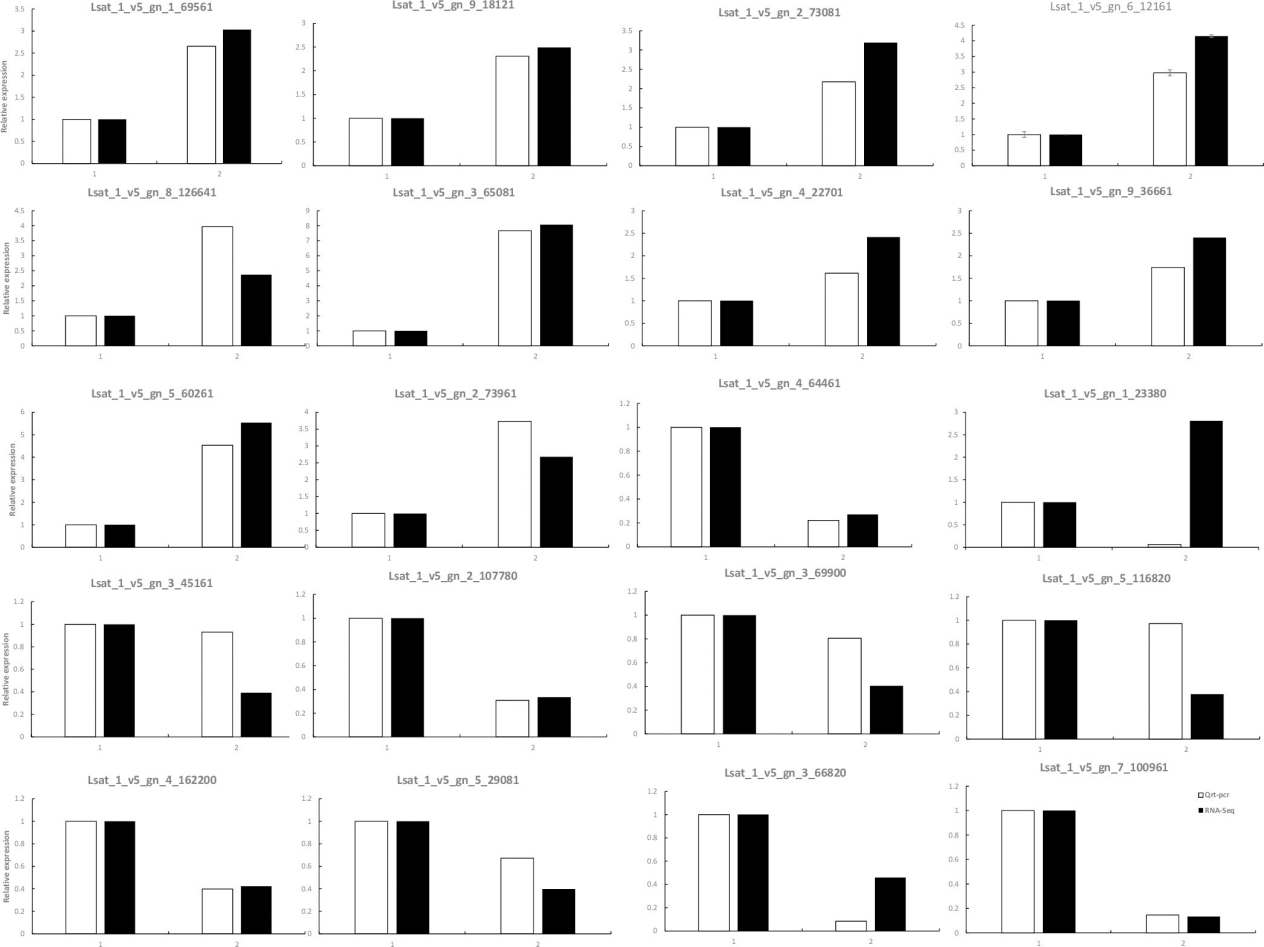
Verification of differentially expressed genes by RT-qPCR. Twenty genes were chosen for RT-qPCR validation. The white and black bars represent the relative expression levels of each gene in the control and high-temperature groups, as detected by RT-qPCR and RNA-Seq, respectively. To plot the RNA-Seq data, gene expression in the control group was set to be the same as that observed by RT-qPCR, and relative expression in the high-temperature group was calculated using the fold-change detected by RNA-Seq. The bars represent the standard deviation (n = 3); 1 represents the control temperature, and 2 represents the high temperature. Asterisks indicate that the gene transcriptions are significantly different between control and treatment group (unpaired t test, P< 0.05).
