# Supplementary figures and images for: Transcriptomic analysis reveals the roles of gibberellin-regulated genes and transcription factors in regulating bolting in lettuce (Lactuca sativa L.)
Source: PLoS One. 2018 Feb 7;13(2):e0191518. doi: 10.1371/journal.pone.0191518 (PMC5802892; doi:10.1371/journal.pone.0191518)

# Down Regulated

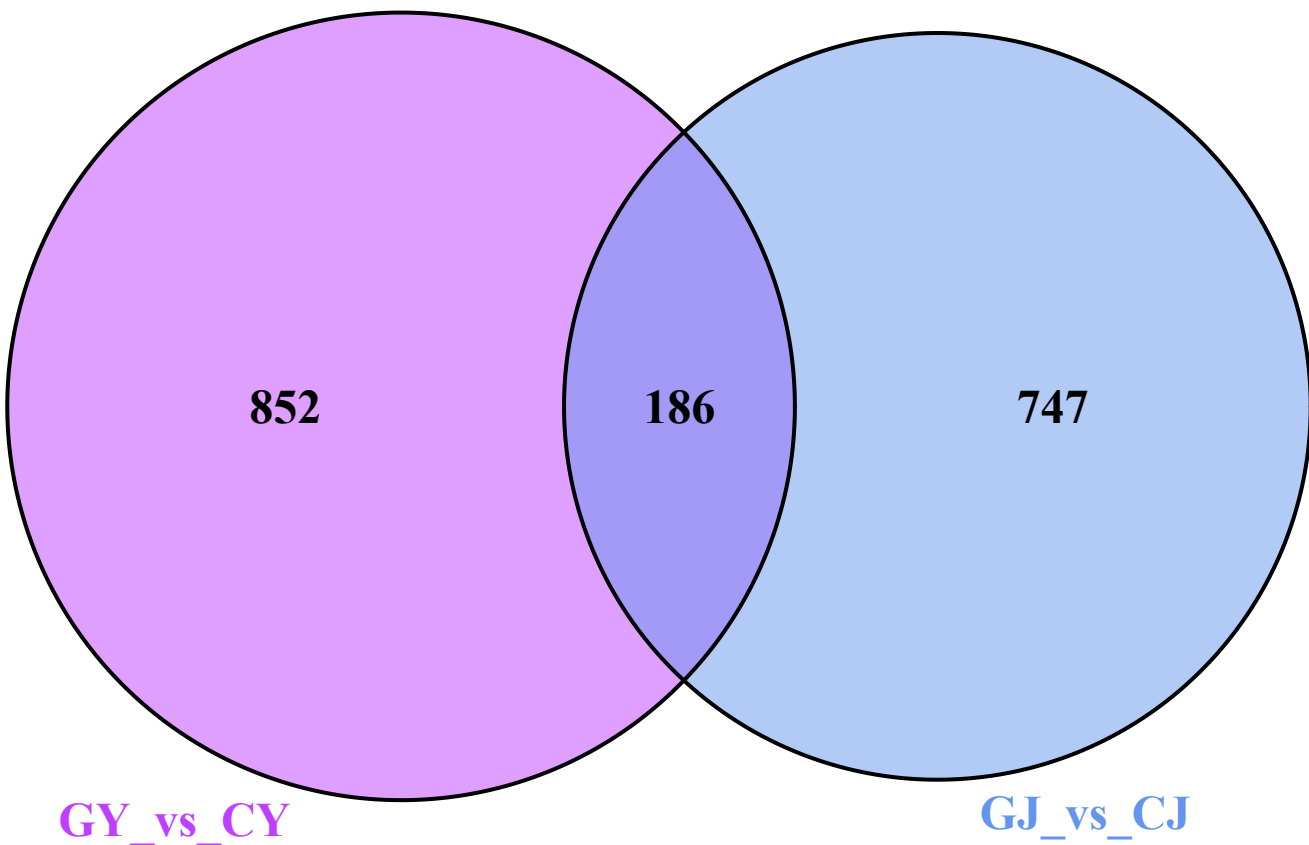

Supplement: S1 Fig — GY and CY represented leaves of high-temperature group and control group. GJ and CJ represented the stem tip of high temperature group and control group, respectively. (PDF) [file pone.0191518.s001.pdf]

# Up Regulated

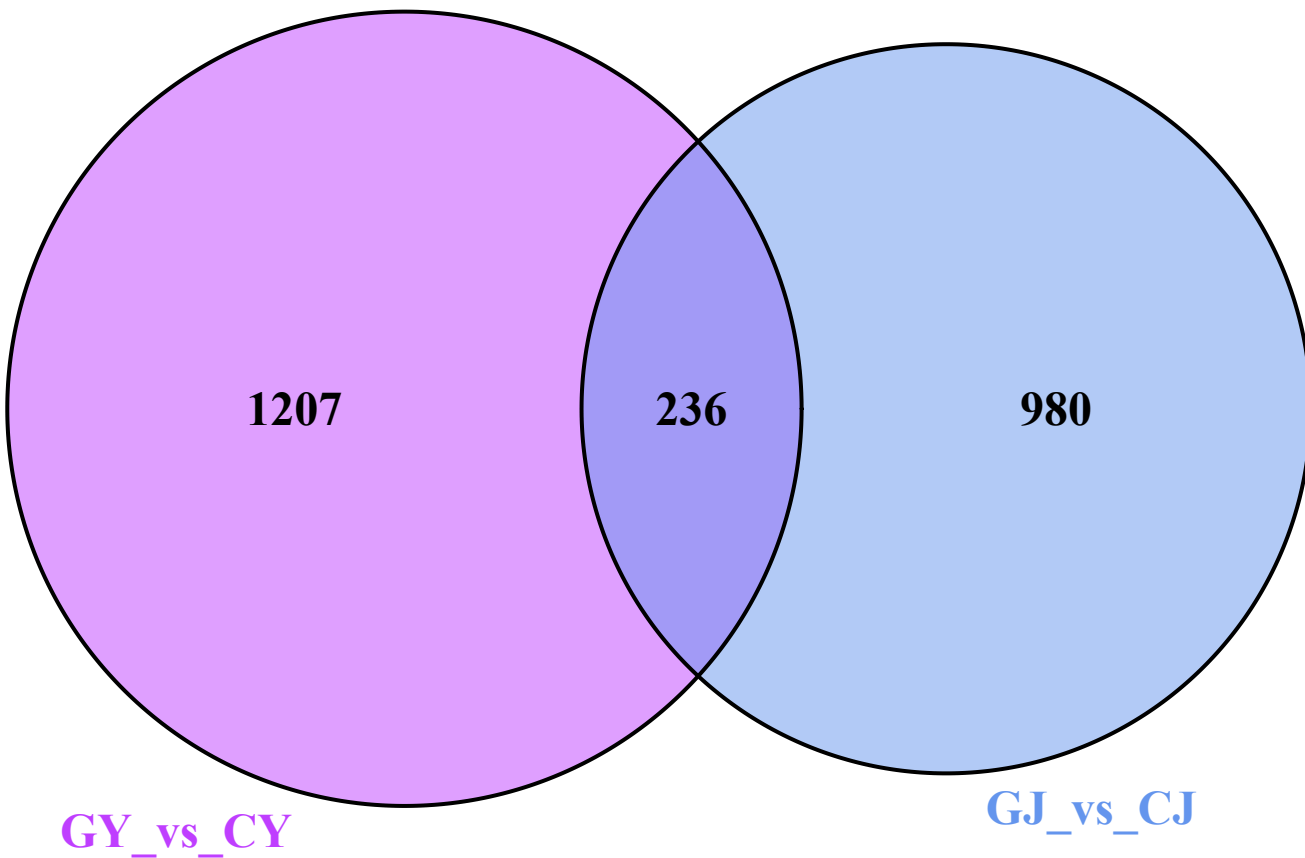

Supplement: S2 Fig — GY and CY represented leaves of high-temperature group and control group. GJ and CJ represented the stem tip of high temperature group and control group, respectively. (PDF) [file pone.0191518.s002.pdf]
